# Comparison of Fibrinogen Concentrate and Cryoprecipitate on Major Thromboembolic Events after Living Donor Liver Transplantation

**DOI:** 10.3390/jcm12237496

**Published:** 2023-12-04

**Authors:** Jae-Hwan Kim, Kyoung-Sun Kim, Hye-Mee Kwon, Sung-Hoon Kim, In-Gu Jun, Jun-Gol Song, Gyu-Sam Hwang

**Affiliations:** Laboratory for Cardiovascular Dynamics, Department of Anesthesiology and Pain Medicine, Asan Medical Center, University of Ulsan College of Medicine, Seoul 05505, Republic of Korea; jaehwankim@amc.seoul.kr (J.-H.K.); kyoungsun.kim@amc.seoul.kr (K.-S.K.); hyemee.kwon@amc.seoul.kr (H.-M.K.); shkimans@amc.seoul.kr (S.-H.K.); igjun@amc.seoul.kr (I.-G.J.); kshwang@amc.seoul.kr (G.-S.H.)

**Keywords:** fibrinogen, liver transplantation, mortality, thromboembolism

## Abstract

(1) Background: Liver transplantation (LT) is associated with significant hemorrhage and massive transfusions. Fibrinogen replacement has a key role in treating massive bleeding during LT and hypofibrinogenemia is treated by fibrinogen concentrate or cryoprecipitate. However, these two products are known to be associated with major thromboembolism events (MTEs). We aimed to compare the effect of fibrinogen concentrate and cryoprecipitate on MTEs in living donor LT (LDLT) recipients. (2) Methods: We analyzed 206 patients who underwent LDLT between January 2021 and March 2022. The patients were divided into two groups according to fibrinogen concentrate or cryoprecipitate use. We compared the incidence of MTEs between the two groups. In addition, we performed multiple logistic regression analyses to identify the risk factors for MTEs. (3) Results: There was no significant difference in the incidence of MTEs (16 [14.7%] vs. 14 [14.4%], *p* = 1.000) between the cryoprecipitate group and fibrinogen concentrate group. In the multivariate analysis, cryoprecipitate (OR 2.09, 95%CI 0.85–5.11, *p* = 0.107) and fibrinogen concentrate (OR 2.05, 95%CI 0.82–5.12, *p* = 0.126) were not significantly associated with MTEs. (4) Conclusions: there was no significant difference in the incidence of MTEs between cryoprecipitate and fibrinogen concentrate in LDLT recipients.

## 1. Introduction

Patients undergoing liver transplantation (LT) are at risk of hemorrhage and receive massive transfusions if needed [1]. Hemostatic and coagulopathy related to cirrhotic liver disease are also known to cause massive bleeding in LT [2]. Fibrinogen has a key role in hemostasis and activates platelet aggregation by binding to glycoprotein IIb and IIIa receptors on platelet surfaces [3]. However, intraoperative fibrinogen levels are reduced because of hemorrhage followed by resuscitation with fluids and fibrinogen-poor blood products [4]. In cases of significant hemorrhage and hypofibrinogenemia, guidelines recommend treatment with either cryoprecipitate or fibrinogen concentrate [5].

Although cryoprecipitate and fibrinogen concentrate are used to treat hypofibrinogenemia, there is concern about thromboembolic risk in using two products. Thromboembolic complications including hepatic artery thrombosis are associated with a high rate of mortality and graft loss in LT [6,7].

In particular, cryoprecipitate is considered to have a higher thromboembolic risk than fibrinogen concentrate because cryoprecipitate is a non-purified product with platelet microparticles, fibronectin, factor VIII, and von Willebrand factor [8]. Previous studies on patients undergoing cardiac surgery have reported that the two products have similar thromboembolic risks [5,9]. However, a direct comparison of thromboembolic events between the two products in living donor LT (LDLT) is lacking in the literature.

Therefore, the aim of this study was to evaluate the effect of cryoprecipitate and fibrinogen concentrate on major thromboembolic events (MTEs) in patients undergoing LDLT. In addition, the incidence of 30-day major adverse cardiovascular events (MACEs), and 1-year graft failure and mortality were also compared between the two groups.

## 2. Materials and Methods

### 2.1. Study Design and Population

We analyzed LT recipients who underwent LDLT at our center between January 2021 and March 2022. Those who acquired hypofibrinogenemia were included and the following patients were excluded: age < 18 years, history of allergic reaction to fibrinogen concentrate or cryoprecipitate, did not receive any blood products transfusion, underwent multi-organ transplantation surgery, or those with missing data.

The Institutional Review Board of Asan Medical Center (protocol no. 2022-0724) approved the study design and waived the requirement for written informed consent based on the retrospective nature of the study. The research protocol followed the ethical guidelines of the 1975 Declaration of Helsinki reflected in the prior approval of the institution’s human research committee. Each transplantation procedure was evaluated and approved by the local authorities and the Korean Network for Organ Sharing affiliated with the Ministry of Health and Welfare of the Republic of Korea.

### 2.2. Data Collection

Patient demographics and perioperative variables were collected using the electronic medical records of our institution. Patient characteristics included age, sex, diabetes mellitus (DM), hypertension (HTN), chronic kidney disease (CKD), coronary artery disease (CAD), cerebrovascular accident (CVA), Model for End-stage Liver Disease score (MELD-Na score), Child–Turcotte–Pugh score (CTP score), and causes for liver transplantation (i.e., HBV-related liver cirrhosis, HCV-related liver cirrhosis, alcoholic liver cirrhosis, hepatocellular carcinoma). Intraoperative laboratory values included hemoglobin, platelet, international normalized ratio (INR), creatinine, total bilirubin, albumin, aspartate transaminase (AST), alanine transaminase (ALT), and sodium. Variables related to intraoperative transfusion included massive transfusion, unit of transfused packed red blood cell (pRBC), fresh frozen plasma (FFP), platelet apheresis, cryoprecipitate, fibrinogen concentrate, baseline fibrinogen level in plasma, maximum amplitude at 10 min and maximum clot firmness in FIBTEM, and fibrinogen level in plasma after treatment protocol. Massive transfusion was defined as the use of more than 10 units of PRBCs within 24 h or more than 4 units within 1 h during surgery.

### 2.3. Transfusion Technique

Patients were divided into two groups according to whether they received fibrinogen concentrate or cryoprecipitate. The transfusion criterion for using fibrinogen concentrate or cryoprecipitate is fibrinogen < 80 mg or rotational thromboelastometry (ROTEM, Tem International GmbH, Munich, Germany) FIBTEM-maximum clot firmness (MCF) < 4 mm. Which blood product to use was determined by the anesthesiologist’s discretion and the blood bank’s inventory. Fibrinogen concentrate dose was approximated using the following formula [10]:

Dose = [target FIBTEM-MCF (mm) − current FIBTEM-MCF (mm)] × weight (kg)/140

Fibrinogen content in the cryoprecipitate varies (150 mg–200 mg) depending on the manufacturer and blood donor. The U.S. Food and Drug Administration (FDA) for fibrinogen content in cryoprecipitate requires a minimum of 150 mg per unit [11,12]. Accordingly, we assumed that 1 unit of cryoprecipitate contained 200 mg of fibrinogen, and for a 70 kg adult, 2 g of fibrinogen or 10 units of cryoprecipitate was used because we targeted FIBTEM-MCF (mm) ≥ 8 mm. At our institution, intraoperative laboratory values and ROTEM were measured three times during the preanhepatic, anhepatic, and neohepatic periods.

### 2.4. Primary and Secondary Outcomes

The primary outcome was a composite of an MTE such as portal and hepatic vein thrombosis, hepatic artery thrombosis, intra-cardiac thrombus, pulmonary embolism, and ischemic stroke (by ultrasonography, transesophageal echocardiography, computed tomography) during 30 days after LDLT. Secondary outcomes were 30-day MACE, 1-year graft failure, and 1-year mortality.

A MACE was defined as the composite of postoperative cardiovascular mortality, atrial fibrillation, ventricular arrhythmias, ST-T wave changes with chest tightness, myocardial infarction [13].

### 2.5. Statistical Analysis

Data are presented as mean ± standard deviation, median (interquartile range [IQR]), or number (proportion), as appropriate. We used the chi-squared test or Fisher’s exact test for categorical variables and Student’s *t*-test or Mann–Whitney U test for continuous variables. Multivariable logistic regression analysis was applied to identify the risk factors for MTEs. We performed multiple logistic regression analysis including patients (*n* = 105) who did not receive cryoprecipitate and fibrinogen concentrate but received pRBC or FFP transfusion. All variables with *p* values < 0.1 in the univariate analysis were included in the multivariate analysis by backward elimination. Kaplan–Meier survival curve was used to depict the risk of 1-year mortality and graft failure. The log-rank test was used to evaluate differences between curves. All data were analyzed using SPSS Statistics for Windows, version 22.0 (IBM Corp., Armonk, NY, USA) or R version 3.1.2 (R Foundation for Statistical Computing, Vienna, Austria).

## 3. Results

**Figure 1:**
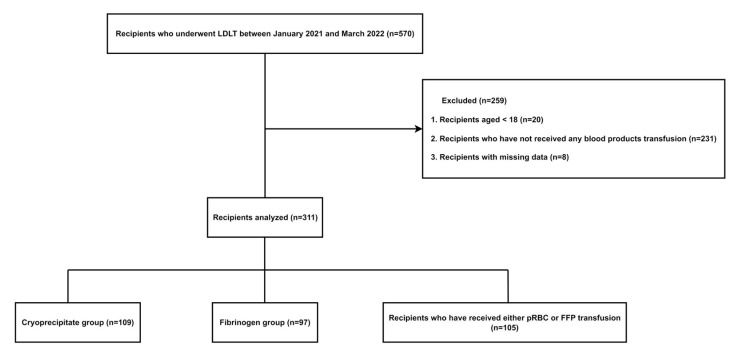
Study flow chart.

Of the 570 patients who underwent LDLT at our institution during the study period, 364 patients were excluded according to the exclusion criteria. In total, 311 patients were divided into the cryoprecipitate group (*n* = 109), fibrinogen concentrate group (*n* = 97), and recipients who received a pRBC or FFP transfusion without cryoprecipitate or fibrinogen concentrate (*n* = 105). (Figure 1)

Table 1 shows the baseline characteristics and perioperative variables of the study patients. The median recipient age was 56 (50.0–62.0) years and 147 (71.4%) were men. Of the 206 recipients, 63 (30.6%) had DM, 45 (21.8%) had HTN, 8 (3.9%) had CKD, 5 (2.4%) had CAD, and 5 (2.4%) had CVA. Alcoholic liver cirrhosis (*n* = 89, 43.2%) was the most common cause of LT, followed by hepatocellular carcinoma (*n* = 80, 38.8%), HBV-related liver cirrhosis (*n* = 80, 38.8%), and HCV-related liver cirrhosis (*n* = 11, 5.3%).

Except for the older age in the fibrinogen group (55.0 vs. 58.0, *p* = 0.046), the two groups did not show significant differences in patient-related variables such as sex, DM, HTN, CKD, CAD, CVA, MELD-Na score, CTP score, and cause for LT. The two groups did not show significant differences in the laboratory variables except for higher preoperative creatinine levels in the cryoprecipitate group (0.85 [0.72–1.15] vs. 0.78 [0.61–0.98], *p* = 0.036). With regard to the intraoperative variables, the two groups did not show significant differences in the operation time (*p* = 0.816), total use of crystalloid (*p* = 0.187), and total use of synthetic colloid (*p* = 0.190). The fibrinogen group had more urine output than the cryoprecipitate group (1570.0 [1010.0–2350.0] vs. 2000.0 [1400.0–2690.0], *p* = 0.006).

Table 2 shows the variables for the intraoperative transfusion and fibrinogen levels and ROTEM values before and after intervention. The intraoperative transfusion variables (massive transfusion, pRBC, FFP, platelet apheresis) were not significantly different between the two groups. The baseline fibrinogen levels of the cryoprecipitate group and the fibrinogen concentrate group were 75.0 (60.0–86.0) and 78.0 (62.0–96.0) (*p* = 0.206), respectively. Also, the results of the baseline MA10 (4.0 [3.0–6.0] vs. 4.0 [3.0–6.0], *p* = 0.521) and MCF (4.0 [3.0–7.0] vs. 5.0 [4.0–7.0], *p* = 0.479) of the FIBTEM and fibrinogen levels after intervention (97.0 [78.0–120.0] vs. 100.0 [81.0–116.0], *p* = 0.838) were not significantly different between the two groups. However, in ROTEM, the fibrinogen group had a significantly higher MA 10 (4.0 [3.0–6.0] vs. 5.0 [3.0–6.0], *p* = 0.033) and MCF (4.0 [3.0–6.0] vs. 5.0 [4.0–7.0], *p* = 0.019) after intervention. (Table 2).

### 3.1. Primary Outcome

There were no significant differences in the incidence of MTEs between the cryoprecipitate group and the fibrinogen concentrate group (16 [16.7%] vs. 14 [14.4%], *p* = 1.000; Table 3). MTEs occurred in three cases, which were hepatic artery thrombosis in two patients (1.8%) in the cryoprecipitate group and ischemic stroke in one patient (1.0%) in the fibrinogen group. There were no cases of intra-cardiac thrombus or pulmonary embolism. Multivariate analysis demonstrated that the duration of surgery (hour, OR 1.22, 95% CI 1.04–1.44, *p* = 0.014) was significantly associated with an MTE (Table 4).

To determine the impact of cryoprecipitate and fibrinogen concentrate on thromboembolism, we compared the incidence of MTEs in patients (*n* = 105) who did not receive cryoprecipitate and fibrinogen concentrate but received either a pRBC or FFP transfusion with patients (*n* = 206) who received either cryoprecipitate or fibrinogen concentrate during LDLT. There was no statistical difference in MTEs between the two groups (Supplemental Table S1).

### 3.2. Secondary Outcomes

There were no significant differences in the incidence of 30-day MACE (24 [22.0%] vs. 13 [13.4%], *p* = 0.154), 1-year mortality (10 [9.2%] vs. 7 [7.2%], *p* = 0.798), and 1-year graft failure (16 [14.7%] vs. 8 [8.3%], *p* = 0.223) between the cryoprecipitate group and the fibrinogen concentrate group (Table 3).

**Figure 2:**
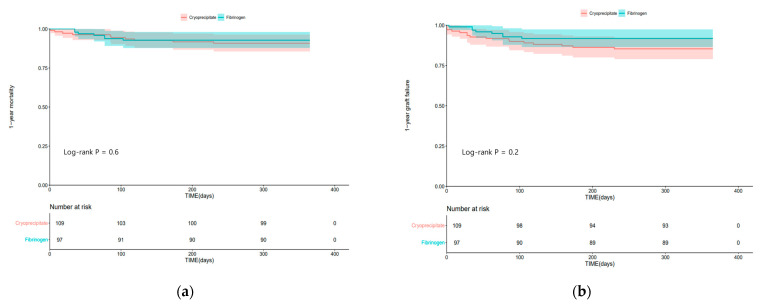
Kaplan–Meier curves of (**a**) 1-year mortality; (**b**) 1-year graft failure in the cryoprecipitate group and fibrinogen concentrate group.

Figure 2 shows the Kaplan–Meier curves of 1-year mortality and 1-year graft failure in the two groups. One-year mortality (log-rank test; *p* = 0.6) and graft failure (log-rank test; *p* = 0.2) were not significantly different between the two groups.

## 4. Discussion

In this retrospective study, we found that there was no significant difference in MTEs between LDLT patients using cryoprecipitate and those using fibrinogen concentrate. Moreover, there were no significant differences in the incidences of 30-day MACE, 1-year graft failure, and mortality between the two groups.

Fibrinogen is a plasma glycoprotein synthesized in the liver. It transforms into fibrin by thrombin, playing a crucial role in clot formation, platelet activation, and aggregation [14]. While cryoprecipitate and fibrinogen concentrate are both plasma-derived, fibrinogen concentrate has a standardized concentration, leading to a predictable hemostatic effect; moreover, fibrinogen concentrate is purified, pasteurized, and filtered, which results in lower risks of viral transmission and immunological transfusion reaction [8,9]. Additionally, fibrinogen concentrate is easily reconstituted in sterile water and has a low administration volume and short administration time; after reconstitution, fibrinogen concentrate has a long shelf life of up to 24 h, thus reducing wastage [8]. Cryoprecipitates are allogenic blood products that are non-purified and contain various coagulation factors in addition to fibrinogen such as factor VIII, factor XIII, and von Willebrand factor [11]. The variability of fibrinogen contents in cryoprecipitate hinders an accurate prediction of its hemostatic effect; however, various coagulation factors have a positive impact on hemostasis in patients with hemodilution or massive blood loss [8,11].

Despite advances in surgical techniques, understanding of the pathophysiology of coagulation in end-stage liver disease patients, and point-of-care treatment, LT is still expected to cause massive bleeding and require a massive transfusion [1]. Acquired hypofibrinogenemia is followed by fluid resuscitation and fibrinogen-poor blood transfusion in surgery, and dysfibrinogenemia is common in LT recipients [15]. In our study, a massive transfusion was observed whether fibrinogen concentrate or cryoprecipitate was administered for acquired hypofibrinogenemia.

Before treatment for acquired hypofibrinogenemia, the baseline plasma fibrinogen levels (mg/dL) for the two groups were 75.0 in the cryoprecipitate group and 78.0 in the fibrinogen concentrate group. The American Society of Anesthesiologists task force for perioperative blood management recommends fibrinogen replacement in patients with bleeding when the plasma fibrinogen level is less than 80–100 mg/dL [16]. In our study, although there were no differences in the baseline fibrinogen levels, MA10, and MCF of FIBTEM between the two groups, the fibrinogen group showed a significantly higher MA 10 (4.0 [3.0–6.0] vs. 5.0 [3.0–6.0], *p* = 0.033) and MCF (4.0 [3.0–6.0] vs. 5.0 [4.0–7.0], *p* = 0.019) in ROTEM after intervention (Table 2). However, no significant difference was found in the fibrinogen levels between the two groups after intervention. In a systematic review comparing cryoprecipitate and fibrinogen concentrate in bleeding patients, it was also reported that there was no significant difference in the increased plasma fibrinogen levels between the two groups after intervention [17]. To assess clot strength, FIBTEM MCF in ROTEM is employed. In a study utilizing a trauma-induced coagulopathy model, it was found that after administration, fibrinogen concentrate resulted in a stronger FIBTEM MCF value compared to cryoprecipitate [18]. However, in a randomized controlled trial conducted by Galas et al. in pediatric cardiac surgery, there was no significant difference in FIBTEM MCF between cryoprecipitate and fibrinogen concentrate after intervention [9]. We presume that this discrepancy in the viscoelastic coagulation test is due to the variability of fibrinogen levels in cryoprecipitate [11,12]. In our study, we assumed that one unit of cryoprecipitate contained 200 mg of fibrinogen; however, as the volume of one unit of cryoprecipitate varies from 15 mL to 20 mL, the fibrinogen content also varies from 150 mg to 200 mg, in which the fibrinogen concentrate is standardized. Our study also demonstrated results similar to a previous study. However, considering the weak coagulation balance in patients with ESLD and the occurrence of massive bleeding during surgery and hemodilution, we believe that further clinical research is needed in this context.

The currently recommended treatment for hypofibrinogenemia is fibrinogen concentrate or cryoprecipitate [19]. Fibrinogen administration in LT for hypofibrinogenemia reduces surgical bleeding [20]; however, fibrinogen concentrate and cryoprecipitate carry thromboembolic risks. A previous study reported that cryoprecipitate is associated with a major thromboembolic risk [6], but did not make a direct comparison between cryoprecipitate and fibrinogen concentrate, and there are only a few studies comparing the thromboembolic risk of the two products. Recent randomized trials of adult [5] and children [9] patients undergoing cardiac surgery demonstrated that there was no significant difference in the thromboembolic risk between fibrinogen concentrate and cryoprecipitate. A systematic review also showed that the two products had no significant difference in thromboembolic risk and did not mention whether one product was superior to the other [17]. Especially in patients with end-stage liver disease, the decrease in both procoagulant and anticoagulant factors can lead to a weak rebalanced hemostasis, making them susceptible to both hemorrhagic and thrombotic complications [21]. Notably, several studies have suggested that fibrinogen supplementation does not increase thromboembolic risk [20,22]. This supports our current findings that fibrinogen replacement using cryoprecipitate or fibrinogen concentrate does not increase thromboembolic risk, emphasizing the safety of fibrinogen administration in the setting of coagulopathy during LT.

In our study, the incidence of any adverse outcomes including 1-year mortality was not significantly different between the two groups. Several studies comparing the two products were conducted in various clinical settings, such as cardiac surgery [5,9,23], obstetric bleeding [24], and trauma [25] in which acquired hypofibrinogenemia frequently appears. A recent randomized clinical trial on patients undergoing cardiac surgery demonstrated that no statistically significant difference was found in the mortality rate between the fibrinogen concentrate group and the cryoprecipitate group [5]. In addition, postoperative mortality in pediatric cardiac surgery was also not significantly different between the two products [9,23].

In our study, there were no statistically significant differences in the incidence of 1-year graft failure (14.7% vs. 8.3%, *p* = 0.223) and MACE (22.0% vs. 13.4%, *p* = 0.154) between the two groups, although the incidences were numerically higher in the cryoprecipitate group. We speculate that the small sample size of our study might be one of the reasons that a significant difference between the two groups was not found in the secondary outcomes. Moreover, while HBV-related LC is the most common cause of LT in South Korea [26], alcoholic LC was the most common etiology in our study patients. This suggests that alcoholic LC and HCC are accompanied by other etiologies including viral hepatitis. Further studies with larger sample sizes are needed to compare the incidences of graft failure and MACE between these two groups in LDLT.

In our study, the duration of surgery was associated with MTEs. In the same manner, T. Maeda et al. demonstrated that the duration of surgery was a risk factor for thrombosis in a multicenter cardiovascular surgery study [27]. Since thromboembolism in LT has multifactorial causes, further studies are needed.

This study has several limitations. Firstly, as our study was retrospective and based on a single center, unmeasured confounding factors may exist. Secondly, as our study only included patients undergoing LT with hypofibrinogenemia, these findings may not be generalized to other clinical settings that require fibrinogen replacement. In addressing these limitations, it is necessary to conduct future studies with multicenter studies and a prospective design. A larger sample size will improve the statistical power, aiding in reliable conclusions and revealing subtle associations. In addition, exploring subgroups, including disease severity and treatment regimens, is crucial for in-depth insights. Moreover, incorporating diagnostic tools in future research will provide a current evaluation of fibrinogen replacement therapy’s effect on thromboembolic risks. Despite these limitations, our data are meaningful in that the outcomes of fibrinogen concentrate and cryoprecipitate in patients undergoing LDLT were directly compared.

In conclusion, there was no significant difference in MTEs between LDLT patients receiving fibrinogen concentrate and those receiving cryoprecipitate. Fibrinogen concentrate may be used as an alternative to cryoprecipitate in the treatment of acquired hypofibrinogenemia in LDLT.

## Figures and Tables

**Table 1:** Baseline characteristics of the study population.

	Cryoprecipitate Group (*n* = 109)	Fibrinogen Group (*n* = 97)	Total (*n* = 206)	*p*-Value
Demographic data				
Age	55.0 (48.0–61.0)	58.0 (52.0–63.0)	56.0 (50.0–62.0)	0.046
Sex, male	75 (68.1)	72 (74.2)	147 (71.4)	0.481
BMI	22.9 (20.2–26.0)	23.7 (21.3–26.9)	23.2 (20.6–26.4)	0.101
Diabetes	32 (29.4)	31 (32.0)	63 (30.6)	0.800
Hypertension	23 (21.1)	22 (22.7)	45 (21.8)	0.916
CKD	5 (4.6)	3 (3.1)	8 (3.9)	0.847
CAD	3 (2.8)	2 (2.1)	5 (2.4)	1.000
CVA	3 (2.8)	2 (2.1)	5 (2.4)	1.000
MELD-Na score	18.0 (10.0–27.0)	15.0 (11.0–21.0)	17.0 (10.0–24.0)	0.368
CTP score	9.0 (7.0–11.0)	9.0 (7.0–10.0)	9.0 (7.0–11.0)	0.538
Cause for LT				
HBV LC	43 (39.5)	37 (38.1)	80 (38.8)	0.961
HCV LC	5 (4.6)	6 (6.2)	11 (5.3)	0.842
Alcoholic LC	50 (45.9)	39 (40.2)	89 (43.2)	0.497
HCC	40 (36.7)	40 (41.2)	80 (38.8)	0.600
HCC with HBV	27 (24.8)	23 (23.7)	50 (24.3)	0.989
HCC with HCV	4 (3.7)	6 (6.2)	10 (4.9)	0.521
HCC with Alcoholic LC	7 (6.4)	12 (12.4)	19 (9.2)	0.218
Laboratory variables				
Hemoglobin, g/dL	9.3 (8.2–11.0)	10.2 (8.4–12.0)	9.9 (8.3–11.7)	0.052
Platelet, $10^{9}$/L	59.0 (42.0–88.0)	58.0 (38.0–79.0)	58.0 (38.0–82.0)	0.462
INR	1.49 (1.23–1.86)	1.38 (1.24–1.65)	1.42 (1.23–1.75)	0.110
AST	38.0 (28.0–56.0)	37.0 (26.0–52.0)	38.0 (27.0–54.0)	0.347
ALT	21.0 (15.0–34.0)	21.0 (14.0–28.0)	21.0 (15.0–32.0)	0.369
Total bilirubin	2.9 (1.2–7.7)	2.0 (1.2–3.4)	2.2 (1.2–5.2)	0.082
Albumin, g/dL	3.0 (2.6–3.4)	2.9 (2.5–3.4)	2.9 (2.6–3.4)	0.496
Sodium	138.0 (134.0–140.0)	138.0 (134.0–141.0)	138.0 (134.0–141.0)	0.850
Creatinine, mg/dL	0.85 (0.72–1.15)	0.78 (0.61–0.98)	0.82 (0.66–1.06)	0.036
Intraoperative variables				
Operation time, hour	12.5 ± 2.2	12.6 ± 1.9	12.6 ± 2.1	0.816
Crystalloid, mL	6200.0 (4400.0–8900.0)	7200.0 (5200.0–9700.0)	6450.0 (4700.0–9050.0)	0.187
Colloid, mL	3600.0 (2800.0–4800.0)	4000.0 (2800.0–5600.0)	3600.0 (2800.0–5200.0)	0.190
Urine output, mL	1570.0 (1010.0–2350.0)	2000.0 (1400.0–2690.0)	1755.0 (1200.0–2580.0)	0.006

Note: values are expressed as the mean ± SD, number (%), or median (1Q, 3Q). Abbreviations: BMI, body mass index; CKD, chronic kidney disease; CAD, coronary artery disease; CVA, cerebrovascular accident; MELD-Na, Model for End-stage Liver Disease-Sodium; CTP, Child–Turcotte–Pugh; LT, liver transplantation; LC, liver cirrhosis; HCC, hepatocellular carcinoma; INR, international normalized ratio; AST, aspartate aminotransferase; ALT, alanine aminotransferase.

**Table 2:** Details of intraoperative transfusion and intervention.

	Cryoprecipitate Group (*n* = 109)	Fibrinogen Group (*n* = 97)	Total (*n* = 206)	*p*-Value
Intraoperative transfusion				
pRBC (unit)	10.0 (6.0–18.0)	10.0 (5.0–17.0)	10.0 (6.0–18.0)	0.757
FFP (unit)	10.0 (4.0–14.0)	10.0 (6.0–18.0)	10.0 (5.0–16.0)	0.461
Platelet apheresis (unit)	1.0 (0.0–1.0)	1.0 (0.0–1.0)	1.0 (0.0–1.0)	0.990
Fibrinogen (g)	0.0 (0.0–0.0)	2.0 (2.0–4.0)	0.0 (0.0–2.0)	<0.001
Cryoprecipitate (unit)	10.0 (10.0–10.0)	0.0 (0.0–0.0)	5.0 (0.0–10.0)	<0.001
Massive transfusion	57 (52.3)	54 (55.7)	111 (53.9)	0.730
Baseline				
Fibrinogen in plasma (mg/dL)	75.0 (60.0–86.0)	78.0 (62.0–96.0)	77.0 (60.0–91.0)	0.206
FIBTEM				
MA 10 (mm)	4.0 (3.0–6.0)	4.0 (3.0–6.0)	4.0 (3.0–6.0)	0.521
MCF (mm)	4.0 (3.0–7.0)	5.0 (4.0–7.0)	5.0 (3.0–7.0)	0.479
After treatment for acquired hypofibrinogenemia				
Fibrinogen in plasma (mg/dL)	97.0 (78.0–120.0)	100.0 (81.0–116.0)	98.0 (80.0–118.0)	0.838
FIBTEM				
MA 10 (mm)	4.0 (3.0–6.0)	5.0 (3.0–6.0)	5.0 (3.0–6.0)	0.033
MCF (mm)	4.0 (3.0–6.0)	5.0 (4.0–7.0)	5.0 (3.0–7.0)	0.019
Fibrinogen administered				
Preanhepatic	21 (19.3)	25 (25.8)	46 (22.3)	1.000
Anhepatic	12 (11.0)	2 (2.1)	14 (6.8)	
Postreperfusion	76 (69.7)	70 (72.2)	146 (70.9)	

Note: values are expressed as median (1Q, 3Q) or number (%). Abbreviations: pRBC, packed red blood cell; FFP, fresh frozen plasma; FIBTEM, assay for tissue factor activation and platelet inhibition; MA 10, maximum amplitude at 10 min; MCF, maximum clot firmness.

**Table 3:** Primary and secondary outcomes.

	Cryoprecipitate Group (*n* = 109)	Fibrinogen Group (*n* = 97)	Total (*n* = 206)	*p*-Value
MTE	16 (14.7)	14 (14.4)	30 (14.6)	1.000
Portal and hepatic vein thrombosis	14 (12.8)	13 (13.4)	27 (13.1)	1.000
Hepatic artery thrombosis	2 (1.8)	0 (0)	2 (1.0)	0.529
Ischemic stroke	0 (0)	1 (1.0)	1 (0.5)	0.953
30-day MACE	24 (22.0)	13 (13.4)	37 (18.0)	0.154
1-year mortality	10 (9.2)	7 (7.2)	17 (8.3)	0.798
1-year graft failure	16 (14.7)	8 (8.3)	24 (11.7)	0.223

Note: values are presented as number (%). Abbreviations: MTE, major thromboembolic event; MACE, major adverse cardiovascular event.

**Table 4:** Multivariate logistic regression analyses of major thromboembolic events.

	Univariate Analysis			Multivariate Analysis		
	OR	95% CI	*p* Value	OR	95% CI	*p* Value
Age (yr)	0.99	0.96–1.02	0.540			
Male sex	2.71	1.09–6.92	0.031	2.43	0.97–6.11	0.058
Diabetes	1.85	0.92–3.71	0.083	1.79	0.88–3.66	0.110
Hypertension	1.36	0.64–2.89	0.427			
Coronary artery disease	1.32	0.28–6.21	0.723			
Cerebral vascular disease	1.20	0.14–10.27	0.866			
Chronic kidney disease	0.64	0.08–5.13	0.677			
Massive transfusion	1.38	0.69–2.73	0.358			
MELD-Na score	0.99	0.95–1.03	0.678			
Duration of surgery (hour)	1.22	1.05–1.43	0.012	1.22	1.04–1.44	0.014
Cause for LT						
HBV LC	0.86	0.43–1.71	0.662			
HCV LC	3.09	0.92–10.41	0.068			
Alcoholic LC	0.95	0.47–1.91	0.881			
HCC	1.53	0.77–3.01	0.223			
Blood products						
^a^ No transfusion	(reference)					
Cryoprecipitate	2.09	0.85–5.11	0.107			
Fibrinogen concentrate use	2.05	0.82–5.12	0.126			

Abbreviations: OR, odds ratio; CI, confidence interval; HCC, hepatocellular cardinoma; MELD-Na, Model for End-stage Liver Disease-Sodium; LT, liver transplantation; LC, liver cirrhosis. ^a^ Patients who did not receive cryoprecipitate and fibrinogen concentrate but received pRBC or FFP transfusion.

## Data Availability

The data presented in this study are available on request from the corresponding author. The data are not publicly available due to privacy.

## Supplementary Materials

The following supporting information can be downloaded at: https://www.mdpi.com/article/10.3390/jcm12237496/s1, Table S1: demographic feature between no transfusion and fibrinogen products transfusion group.
