# Supplementary material for: Comparison of Fibrinogen Concentrate and Cryoprecipitate on Major Thromboembolic Events after Living Donor Liver Transplantation
Source: J Clin Med. 2023 Dec 4;12(23):7496. doi: 10.3390/jcm12237496 (PMC10707065; doi:10.3390/jcm12237496)
Supplement: Supplementary file 1 [file jcm-12-07496-s001.zip › jcm-2726526-supplementary.pdf]

Supplemental Table S1. Demographic feature between no transfusion and fibrinogen products transfusion group

	[a]No transfusion group (N=105)	Fibrinogen / Cryoprecipitate group (N=206)	Total (N=311)	P-value
Demographic data				
Age	57.0 (48.0-62.0)	56.0 (50.0-62.0)	56.0 (49.0-62.0)	0.755
Sex, male	66 (62.9)	147 (71.4)	213 (68.5)	0.162
BMI	23.6 (21.2-25.8)	23.2 (20.6-26.4)	23.4 (20.9-26.3)	0.996
Diabetes	30 (28.6)	63 (30.6)	93 (29.9)	0.814
Hypertension	29 (27.6)	45 (21.8)	74 (23.8)	0.322
CKD	4 (3.8)	8 (3.9)	12 (3.9)	1.000
CAD	8 (7.6)	5 (2.4)	13 (4.2)	0.062
CVA	2 (1.9)	5 (2.4)	7 (2.3)	1.000
MELD-Na score	10.0 (8.0-15.0)	17.0 (10.0-24.0)	14.0 (9.0-21.0)	<0.001
CTP score	7.0 (6.0-9.0)	9.0 (7.0-11.0)	8.0 (6.0-10.0)	<0.001
Cause for LT				
HBV LC	53 (50.5)	80 (38.8)	133 (42.8)	0.066
HCV LC	3 (2.9)	11 (5.3)	14 (4.5)	0.478
Alcoholic LC	29 (27.6)	89 (43.2)	118 (37.9)	0.011
HCC	55 (52.9)	80 (38.8)	135 (43.4)	0.031
Laboratory variables				
Hemoglobin, g/dl	10.4 (9.2-12.1)	9.9 (8.3-11.7)	10.1 (8.6-11.8)	0.012
Platelet, 10^9/L	76.0 (52.0-111.0)	58.0 (38.0-82.0)	65.0 (42.0-92.5)	<0.001
INR	1.18 (1.08-1.35)	1.42 (1.23-1.75)	1.32 (1.16-1.60)	<0.001
AST	33.0 (24.0-49.0)	38.0 (27.0-54.0)	36.0 (26.0-52.0)	0.028
ALT	19.0 (13.0-29.0)	21.0 (15.0-32.0)	20.0 (14.0-31.0)	0.253
Total bilirubin, mg/dl	1.1 (0.7-2.3)	2.2 (1.2-5.2)	1.7 (0.9-3.9)	<0.001
Albumin, g/dl	3.1 (2.7-3.6)	2.9 (2.6-3.4)	3.0 (2.6-3.5)	0.021
Sodium	139.0 (138.0-141.0)	138.0 (134.0-141.0)	139.0 (136.0-141.0)	<0.001
Creatinine, mg/dl	0.74 (0.60-0.92)	0.82 (0.66-1.06)	0.80 (0.63-1.00)	0.019
Intraoperative variables				
Operation time, hour	11.4 (10.1-12.9)	12.3 (11.2-14.0)	12.0 (10.7-13.7)	<0.001
Crystalloid, ml	5700.0 (4500.0-6900.0)	6450.0 (4700.0-9050.0)	6200.0 (4550.0-8400.0)	0.006
Colloid, ml	2800.0 (2000.0-3200.0)	3600.0 (2800.0-5200.0)	3200.0 (2400.0-4400.0)	<0.001
Urine output	1800.0 (1370.0-2460.0)	1755.0 (1200.0-2580.0)	1765.0 (1255.0-2530.0)	0.493
Outcomes				
MTE	8 (7.6)	30 (14.6)	38 (12.2)	0.113
PVT & HVT	8 (7.6)	27 (13.1)	35 (11.3)	0.208
HAT	0 (0)	2 (1.0)	2 (0.6)	0.793
Ischemic stroke	0 (0)	1 (0.5)	1 (0.3)	1.000

	[a]No transfusion group (N=105)	Fibrinogen / Cryoprecipitate group (N=206)	Total (N=311)	P-value
30-day MACE	21 (20.0)	37 (18.0)	58 (18.7)	0.777

Note: Values are expressed as the mean ± SD, number (%), or median (1Q, 3Q).

Abbreviations: BMI, body mass index; CKD, chronic kidney disease; CAD, coronary artery disease; CVA, cerebrovascular accident; MELD-Na, Model for End-stage Liver Disease-Sodium; CTP, Child-Turcotte-Pugh; LT, liver transplantation; LC, liver cirrhosis; HCC, hepatocellular carcinoma; INR, international normalized ratio; AST, aspartate aminotransferase; ALT, alanine aminotransferase; MTEs, major thromboembolic events; PVT, portal vein thrombosis; HVT, hepatic vein thrombosis; HAT, hepatic artery thrombosis; MACE, Major adverse cardiovascular events

[a] Patients who did not receive cryoprecipitate and fibrinogen concentrate but received pRBC or FFP transfusion
